# Growth of a self-assembled monolayer decoupled from the substrate: nucleation on-command using buffer layers

**DOI:** 10.3762/bjnano.11.113

**Published:** 2020-09-01

**Authors:** Robby Reynaerts, Kunal S Mali, Steven De Feyter

**Affiliations:** 1 Department of Chemistry, Division of Molecular Imaging and Photonics, KU Leuven, Celestijnenlaan 200F, B-3001 Leuven, Belgium

**Keywords:** benzoic acid, nucleation, self-assembly, solution–solid interface, substrate effect

## Abstract

Structural polymorphism is ubiquitous in physisorbed self-assembled monolayers formed at the solution–solid interface. One of the ways to influence network formation at this interface is to physically decouple the self-assembled monolayer from the underlying substrate thereby removing the influence of the substrate lattice, if any. Here we show a systematic exploration of self-assembly of a typical building block, namely 4-tetradecyloxybenzoic acid at the 1-phenyloctane–graphite interface in the presence and in the absence of a buffer layer formed by a long chain alkane, namely *n*-pentacontane. Using scanning tunneling microscopy (STM), three different structural polymorphs were identified for 4-tetradecyloxybenzoic acid at the 1-phenyloctane–graphite interface. Surprisingly, the same three structures were formed on top of the buffer layer, albeit at different concentrations. Systematic variation of experimental parameters did not lead to any new network in the presence of the buffer layer. We discovered that the self-assembly on top of the buffer layer allows better control over the nanoscale manipulation of the self-assembled networks. Using the influence of the STM tip, we could initiate the nucleation of small isolated domains of the benzoic acid on-command in a reproducible fashion. Such controlled nucleation experiments hold promise for studying fundamental processes inherent to the assembly process on surfaces.

## Introduction

The ability of some molecules to crystalize in more than one type of packing – a property widely known as crystal polymorphism – is not limited to the realm of bulk (3D) crystals but also extends to the 2D world of physisorbed self-assembled monolayers [1–4]. In fact, observation of multiple polymorphic networks, especially for organic monolayers formed at the solution–solid interface, is a more of routine occurrence than an exception. Such structurally diverse monolayers are typically formed on solid substrates such as highly oriented pyrolytic graphite (HOPG), graphene, and metals such as Cu, Ag and Au and have been characterized using scanning probe methods, especially scanning tunneling microscopy (STM) [2]. While the formation of structurally diverse crystalline monolayers provides exciting opportunities for surface modification and also for investigating crystal engineering in 2D [5], predicting 2D polymorphism is often nontrivial. The understanding of this already enigmatic process is further impaired by the nature of the solution–solid interface. A number of factors such as the temperature [6–9], the solvent [10–15], the substrate [16–18] and the concentration of the building block in solution [19–24] are known to influence network formation at the solution–solid interface.

One of the unconventional ways to influence the structure formation at the solution–solid interface involves the use of buffer layers. Such buffer layers typically comprise of monolayers formed by long chain alkanes [25–27] or alkane derivatives such as fatty acids [28–30] or alkylamines [31], especially when graphite is used as the substrate. The rationale here is that given the strong influence of the substrate lattice on the adsorption as well as the self-assembly of typical organic molecules, insertion of another layer in between the assembling building block and the substrate would allow the formation of an alternative polymorph that would not be obtained otherwise. In line with this strategy, self-assembled buffer layers of *n*-pentacontane (***n*-C_50_**) have been used to obtain a previously unknown polymorph of hexakis(*n*-dodecyl)-*peri*-hexabenzocoronene (**HBC-C_12_**) which was not formed when the assembly process was carried out at the *n*-tetradecane–HOPG interface without the buffer layer [26]. Buffer layers of ***n*-C_50_** have also been used to ‘select’ certain polymorphs of a Fréchet dendron based on the symmetry of the underlying alkane layer [27]. Buffer layers of tetratriacontane [25] and tridecylamine [31] were used to template the self-assembly of copper phthalocyanine. Room temperature STM measurements revealed that the adsorption as well as the diffusion of clusters of CuPc molecules was strongly influenced by the symmetry and the structure of the buffer layers. Notably, no self-assembly was observed when CuPc solutions were directly deposited on the HOPG substrate highlighting the role of buffer layers in stabilizing the self-assembled networks [25,31].

Besides their use for influencing structure formation by acting as a physical barrier between the substrate and the assembling moiety, buffer layers are also widely used to study intrinsic electronic properties of functional organic systems such as organic semiconductors [32–33] and films of 1D/2D polymers [34–37] via electronic decoupling. Alkane buffer layers have been employed as efficient electronic decoupling platforms for studying the intrinsic electronic properties of graphene and fullerenes [38]. Apart from alkane derivatives, inorganic systems such as chemisorbed iodine layers [34–37], and ultrathin layers of KCl [39], NaCl [40], CuN [41] and oxides [32,42] have been used. Typically, the ultrathin films of these wide band gap materials act as insulating layers while still allowing electron tunneling through them. Chemisorbed iodine layers have been used as passivating layers on metals such as Au for achieving controlled electrochemical polymerization of thiophene to produce polythiophene nanowires with tunable lengths [36–37].

In the context of their use for controlling surface architectures, insertion of the buffer layers between the assembling moiety and the solid substrate affects the assembly process via the following major avenues: (1) The buffer layers, in principle, offer a new substrate with a different, often lower, symmetry. (2) The buffer layer has a different lattice constant compared to the solid substrate. (3) The adsorption enthalpy of the assembling moiety on the buffer layer is often different (lower) on the buffer layer compared to that on the solid substrate. These factors may lead to different adsorption conformation for initial single molecule adsorption coupled with higher orientational freedom and significantly different (often reduced) lateral corrugation barriers for molecular diffusion on buffer layers compared to that on pristine unmodified solid substrates. The result could be a selection of a polymorph that is otherwise not obtained under ‘normal’ experimental conditions. The decoupling effects could be significant especially for organic building blocks substituted with long alkyl chains because the soft-epitaxy of such chains with the graphite lattice is well established [43–45].

**Figure 1:**
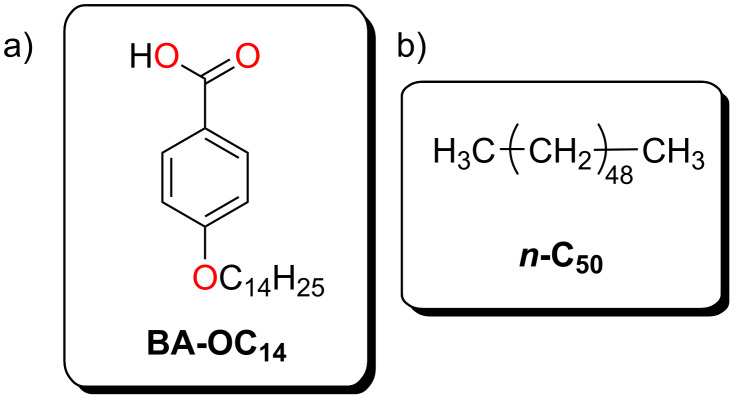
Molecular structures of (a) 4-tetradecyloxybenzoic acid (**BA-OC_14_**) and (b) *n*-pentacontane (***n*-C_50_**) used in this study. Monolayers of ***n*-C_50_** were used as buffer layers to physically decouple the monolayers formed by **BA-OC_14_** from the underlying graphite substrate.

In this contribution, we present a systematic, curiosity-driven study of the self-assembly of a relatively simple building block, namely 4-tetradecyloxybenzoic acid (**BA-OC_14_**, Figure 1a), with and without ***n*-C_50_** (Figure 1b) buffer layers at the 1-phenyloctane–HOPG interface. Our previous investigation into the assembly behavior of this building block revealed that it forms structurally complex monolayers at the 1-phenyloctane–HOPG interface. Despite its simple molecular structure, the networks of **BA-OC_14_** consist of a dense arrangement of hydrogen bonded dimers wherein the molecular columns show periodic kinks along the column axis after every three dimers. The origin of this relatively complex packing was thought to be the specific registry of the alkoxy chains with the substrate lattice that does not allow parallel and straight row packing of dimers due to the steric hindrance of the bulky phenyl groups. The result is a tilted arrangement of the **BA-OC_14_** dimers that show periodic kinks that were thought to arise to maintain the specific registry with the substrate lattice [45]. Considering that the self-assembled network formed by **BA-OC_14_** at the 1-phenyloctane–HOPG interface could possibly represent a substrate lattice-controlled assembly, we set out to address the following questions: (1) Will **BA-OC_14_** form a similar network structure on top of a ***n*-C_50_** buffer layer? (2) Would the self-assembly of **BA-OC_14_** atop the ***n*-C_50_** buffer layer lead to the formation of another polymorph? (3) Does the assembly atop such buffer layers provide better control over our ability to monitor/manipulate dynamic assembly processes? The results and discussion provided below delve into some of these aspects.

## Results and Discussion

### Self-assembly of **BA-OC_14_** without buffer layer

The famous and somewhat provocative quote of McCrone that, “… every compound has different polymorphic forms, and that, in general, the number of forms known for that compound is proportional to the time and money spent in research on that compound.” [46] also applies to on-surface 2D assembly of building blocks. In fact, it is not unusual to observe multiple complex polymorphs for a building block with a seemingly simple molecular structure as demonstrated by the assembly of 4-octadecyloxybenzamide, which was found to form six different network structures [19]. Thus, before carrying out self-assembly experiments with ***n*-C_50_** buffer layers, we systematically studied the concentration dependence of **BA-OC_14_** assembly to comprehensively identify the different structures formed by **BA-OC_14_** at the 1-phenyloctane–HOPG interface. With this exercise, we can have reasonable confidence that any ‘new’ polymorph observed atop buffer layers is indeed a new structure and is not formed as a consequence of slight changes in the solution concentration and thus is also observed in the absence of the buffer layer. To this effect, the concentration dependence of self-assembly was examined within the concentration range of 7.7 × 10^−4^ M to 4.0 × 10^−5^ M. For concentrations below 4.0 × 10^−5^ M, no self-assembly was observed.

**Figure 2:**
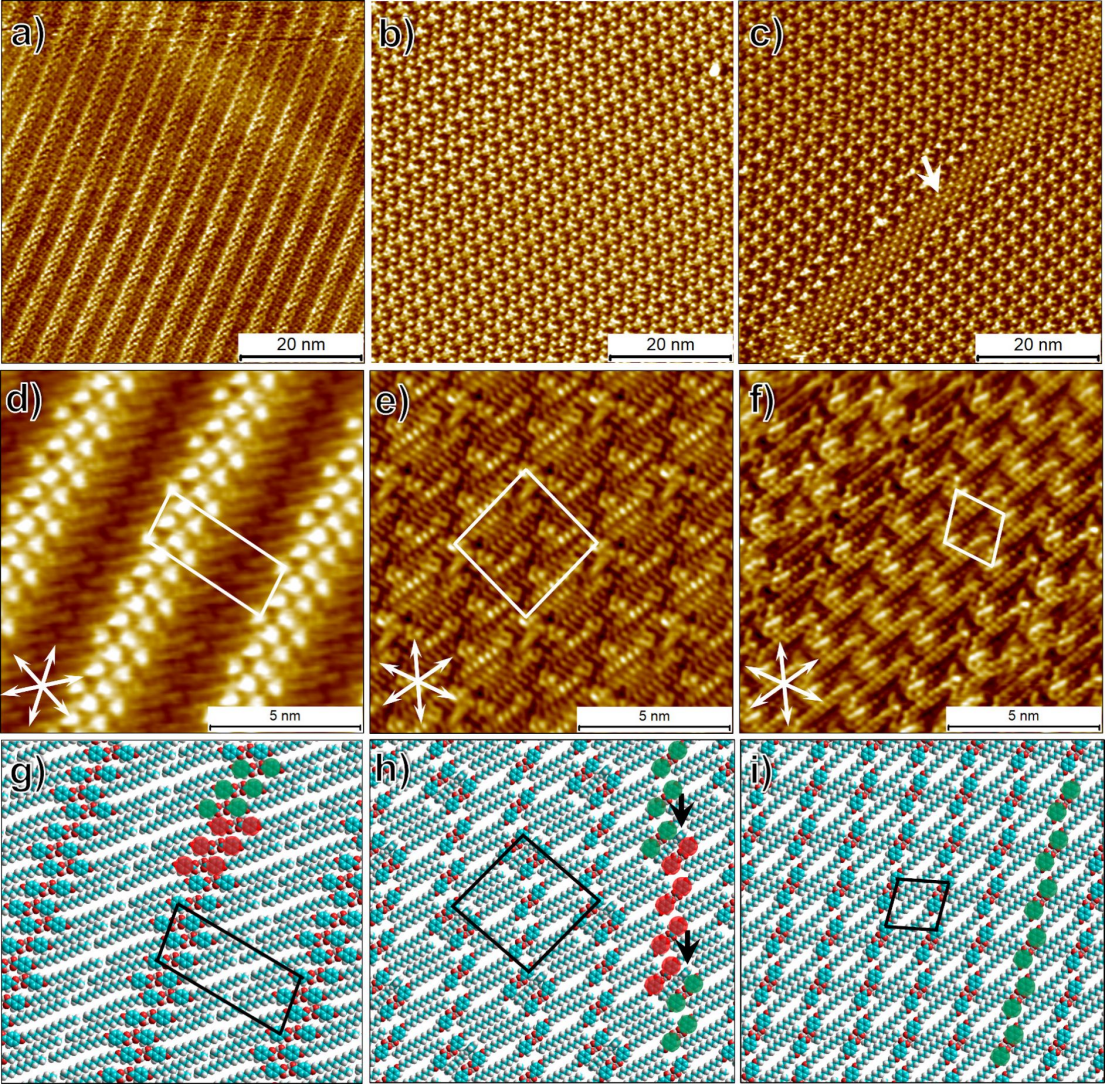
STM images of concentration dependent polymorphs of **BA-OC_14_** formed at the 1-phenyloctane–HOPG interface. (a,b,c) Large scale STM images of polymorph A, B and C, respectively. (d,e,f) High-resolution STM images of polymorph A, B and C, respectively. The white double-headed arrows at the lower left corner of the image represent the three symmetry axes of the graphite lattice. (g,h,i) Molecular models for polymorph A, B and C, respectively. Imaging conditions: (a) *I*_set_ = 70 pA, *V*_bias_ = −1.55 V; (b,c) *I*_set_ = 140 pA, *V*_bias_ = 0.55 V; (d) *I*_set_ = 50 pA, *V* = −1.85 V; (e,f) *I*_set_ = 140 pA, *V*_bias_ = 0.55 V. For additional data, see Figures S1 and S2 in Supporting Information File 1.

Figure 2 shows large-scale and high-resolution STM images of the different polymorphs of **BA-OC_14_** observed at the 1-phenyloctane–HOPG interface together with the proposed molecular models. Polymorph A (Figure 2a,d) is formed exclusively within the concentration range of 7.7 × 10^−4^ M to 1.9 × 10^−4^ M. This network has been reported by us earlier [45]. The high-resolution image provided in Figure 2d shows that the lamellar structure consists of bright blobs corresponding to the benzene rings flanked on either side by relatively darker regions which arise due to the tetradecyloxy chains. The benzene rings always appear in pairs indicating formation of hydrogen bonded dimers. Each lamella shows regular kinks along the lamella propagation direction. The kinks appear after every three hydrogen bonded dimers and the next three dimers are shifted with respect to the previous triplet of dimers (highlighted in red and green color, Figure 2g). The tetradecyloxy chains are oriented at ≈60° with respect to the column axis and are always aligned along one of the main symmetry axes of the graphite lattice. The tetradecyloxy chains in the neighboring lamellae are arranged in a tail-to-tail fashion without any interdigitation. The lamellae make an angle of ≈8° with respect to the nearest symmetry axis of graphite. The unit cell parameters of the network are provided in Table 1 and match with those reported by us earlier [45].

**Table 1:** Unit cell parameters for the benzoic acid polymorphs (A, B and C) observed in this study together with those of ***n*-C_50_** buffer layer. *D*_1_ and *D*_2_ are the molecular densities of the different polymorphs observed on HOPG and atop ***n*-C_50_** buffer layer, respectively. θ_1_ and θ_2_ are the percentage surface coverages of the respective polymorphs at [**BA-OC_14_**] = 9 × 10^−5^ M (on HOPG) and [**BA-OC_14_**] = 3.8 × 10^−4^ M (***n*-C_50_** buffer layer), respectively.

	Unit cell (HOPG)			*D*_1_ (molecules/nm^2^)	θ_1_ (%)	Unit cell (***n*-C_50_** buffer layer)			*D*_2_ (molecules/nm^2^)	θ_2_ (%)
	
	a (nm)	b (nm)	α (°)			a (nm)	b (nm)	α (°)		

A	1.8 ± 0.1	4.4 ± 0.1	81.0 ± 2.7°	0.74	60	1.8 ± 0.1	4.6 ± 0.1	77.9 ± 2.4°	0.71	50
B	3.4 ± 0.1	3.4 ± 0.1	86.9 ± 1.3°	0.68	39	3.6 ± 0.2	3.4 ± 0.1	88.2 ± 4.9°	0.65	49
C	1.7 ± 0.1	1.6 ± 0.1	79.6 ± 1.0 °	0.71	1	1.5 ± 0.1	2.0 ± 0.2	83.6 ± 2.7°	0.68	1
***n*-C_50_**	6.6 ± 0.1	0.5 ± 0.1	90.0 ± 2.0°	–	–	–	–	–	–	–

Lowering the concentration to 9 × 10^−5^ M lead to the formation of two additional networks at the 1-phenyloctane–HOPG interface. Although polymorph A remains the dominant network on the surface with the highest surface coverage (≈60%), another network with a significantly different unit cell (Figure 2b,e and Table 1) was observed. This network, referred hereon as polymorph B, lacks the peculiar bright-dark contrast observed in the STM images of polymorph A. Figure 2e,h shows a high-resolution STM image of polymorph B and the corresponding molecular model, respectively. It can be readily noticed that **BA-OC_14_** molecules still form dimers but the molecules are stacked antiparallel to each other. The dimers show a shift in the propagation direction after every four (parallel) dimers which can be noticed from the proposed molecular model (red and green circles, Figure 2h). Due to this shift, the two (parallel) dimers with a different propagation direction are closer to each other. The molecular model also reveals that the space between four benzene rings may host a molecule of solvent adsorbed edge-on (black arrow, Figure 2h) with respect to the surface. The collection of such four benzene rings together with the co-adsorbed molecule of 1-phenyloctane is often imaged as a single bright feature in large-scale STM images (as evident from Figure 2b). At 9 × 10^−5^ M, the surface coverage of polymorph B was found to be ≈39%.

At 9 × 10^−5^ M, another network, polymorph C, is observed that only slightly differs from polymorph B, and has the lowest surface coverage (≈5%). Polymorph C (white arrow, Figure 2c) tends to appear on the edges of the domains of polymorph B and can be identified by its distinct STM contrast compared to that of polymorph B. Figure 2f,i shows the high-resolution STM image and the proposed molecular model for polymorph C, respectively. It is evident that the network consists of antiparallel dimers similar to those observed in the case of polymorph B, however, the dimers do not show a shift in the propagation direction (green circles, Figure 2i). Polymorph C, despite its low surface coverage was found to be stable to STM scanning and did not transform into other structures within the typical time frame of STM experiments. At concentrations lower than 9 × 10^−5^ M no self-assembly was observed indicating that the concentration limit for **BA-OC_14_** assembly under these conditions is reached.

### Self-assembly of **BA-OC_14_** with ***n*-C_50_** buffer layer

**Figure 3:**
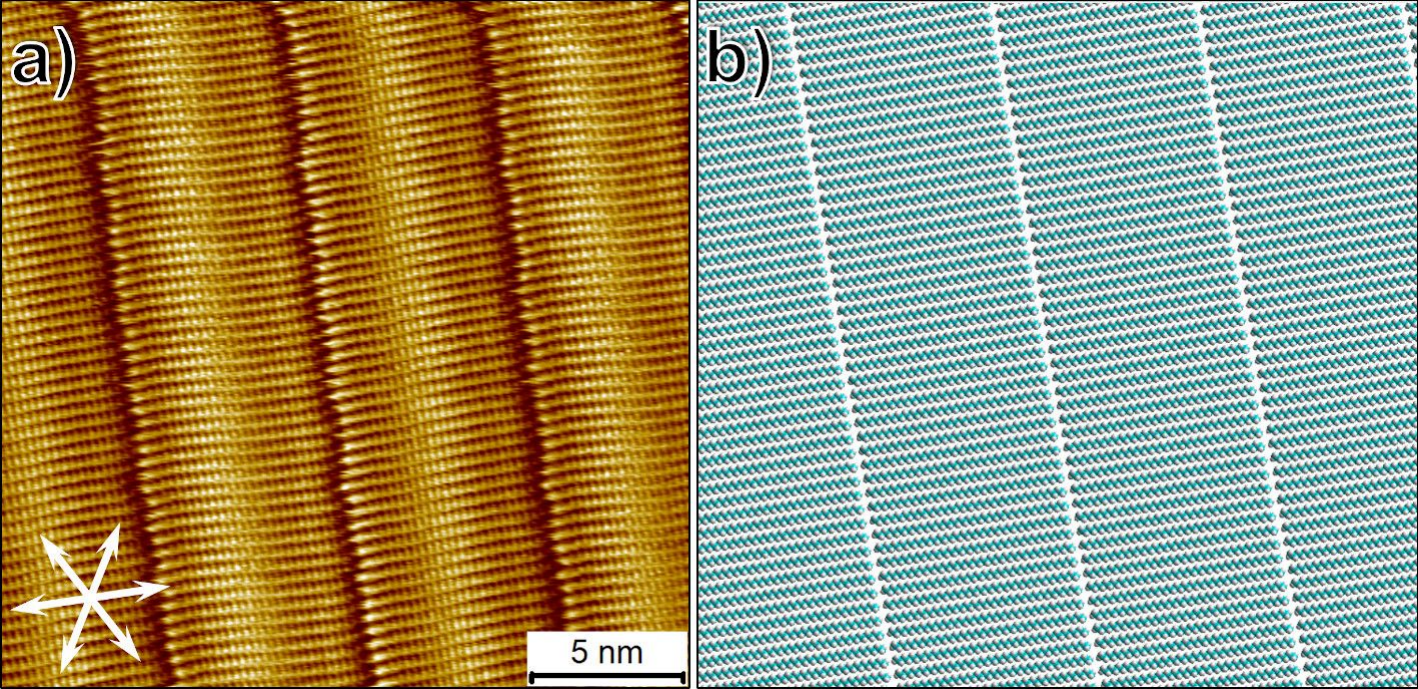
(a) High-resolution STM image of the ***n*-C_50_** monolayer formed at the 1-phenyloctane–HOPG interface. The white double-headed arrows at the lower left corner of the image represent the three symmetry axes of the graphite lattice. (b) Molecular model for the ***n*-C_50_** monolayer. Imaging conditions: *I*_set_ = 200 pA, *V*_bias_ = −0.8 V.

Having confirmed the possibility of the formation of three different polymorphs of **BA-OC_14_** at the 1-phenyloctane–HOPG interface, we turned our attention to the buffer layer experiments. In order to minimize the influence of the domain borders of the buffer layers on the nucleation and the growth of the **BA-OC14** domains, we first optimized the deposition conditions in order to obtain large domains of ***n*-C_50_**. Specifically, we targeted domain sizes of ≥250 nm × 250 nm by carrying out systematic concentration-dependent measurements since lower solution concentrations are known to favor large domain sizes. Annealing of the samples was also carried out. We noticed that at lower solution concentrations, the ***n*-C_50_** monolayers exhibit a highly dynamic behavior with significant reorganization of domains upon repeated scanning with the STM tip (Figure S3 in Supporting Information File 1). Figure 3a,b shows a typical high-resolution STM image of the ***n*-C_50_** buffer layer together with the proposed molecular model for the assembly. ***n*-C_50_** monolayers consist of a lamellar structure in which the alkane molecules are fully extended and are oriented at 90° with respect to the lamella axis. This monolayer structure is identical to that reported earlier [26].

After thoroughly understanding and optimization of the self-assembly behavior of **BA-OC_14_** and ***n*-C_50_** at the 1-phenyloctane–HOPG interface, we moved on to the self-assembly experiments with the buffer layer. Although the buffer layer experiments described below were carried out using sequential deposition of solutions of ***n*-C_50_** and **BA-OC_14_** (in that sequence), we stress that the outcome of the experiments remained the same when carried out using premixed solutions of the two components. Typically, a drop of 1 × 10^−5^ M solution of ***n*-C_50_** was first applied onto a freshly cleaved HOPG surface and the surface was imaged using STM to ensure full coverage of the ***n*-C_50_** monolayer. After this, a drop of **BA-OC_14_** solution was applied and the imaging was resumed. Note that while choosing the concentration of **BA-OC_14_**, the dilution effect arising from the mixing ***n*-C_50_** solution already present on the HOPG surface was taken into account (approximately 50% dilution, since equal volumes of the two solutions were used). Similar precaution was exercised when the premixed solutions were prepared. For **BA-OC_14_** the chosen concentration was the one where its self-assembly behavior on bare HOPG is fully known. Thus, a **BA-OC_14_** concentration of 3.8 × 10^−4^ M was selected because the net concentration after mixing is ≈1.9 × 10^−4^ M where polymorph A was observed at the bare HOPG–1-phenyloctane interface. For ***n*-C_50_**, the chosen optimal concentration was 0.01 mM, because at this concentration we observed reasonably high-average domain size and relatively low dynamics within the ***n*-C_50_** monolayer.

**Figure 4:**
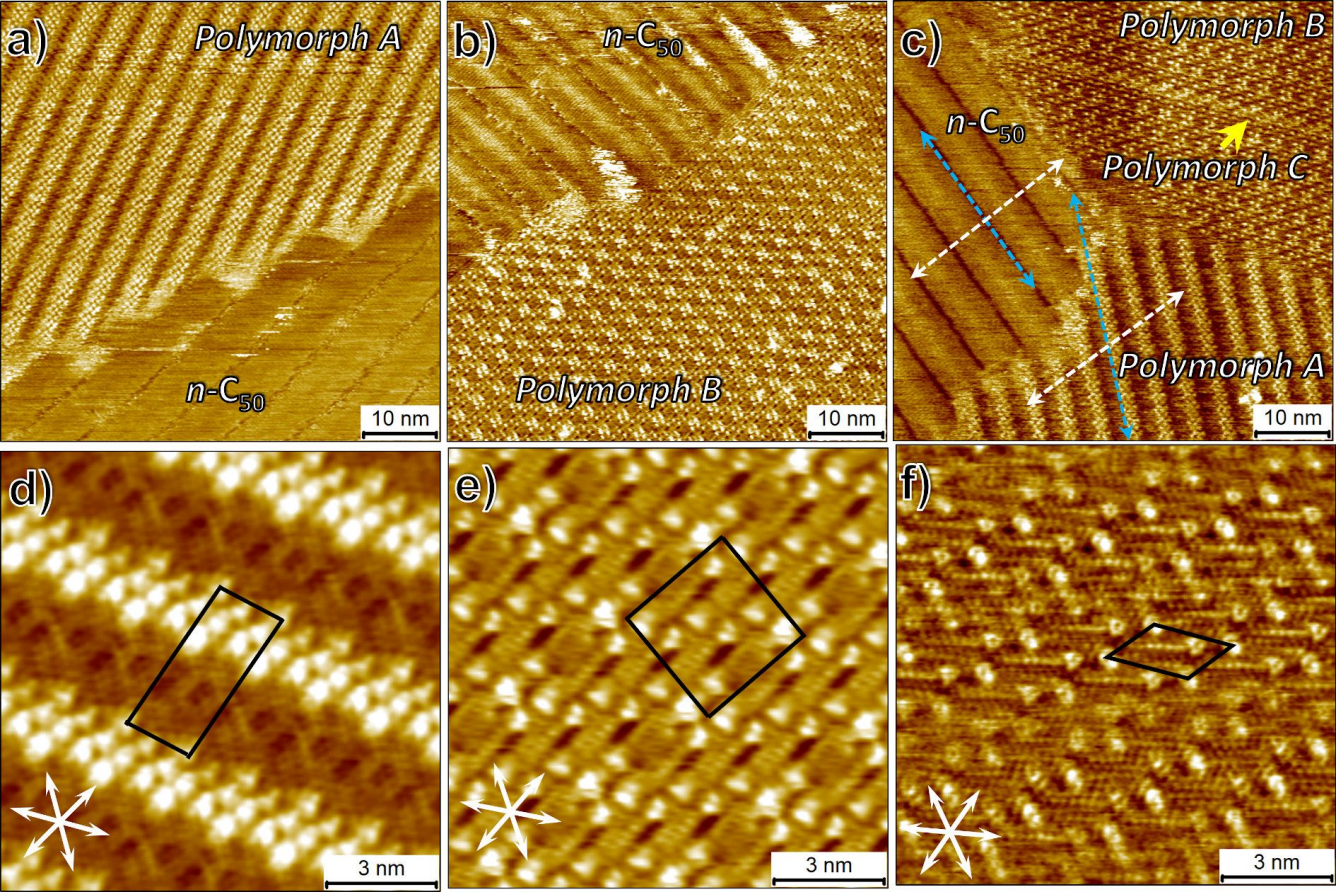
Self-assembled monolayers of **BA-OC_14_** formed on top of the ***n*-C_50_** buffer layer at the 1-phenyloctane–HOPG interface. The experiment was carried out using sequential deposition of the two solutions [**BA-OC_14_**] = 3.8 × 10^−4^ M, [***n*-C_50_**] = 1.0 × 10^−5^ M. (a–c) Representative large scale STM images showing the formation of polymorph A and polymorph B atop the ***n*-C_50_** buffer layer. (d–f) Representative high-resolution images showing the structure of the polymorph A and polymorph B atop the ***n*-C_50_** buffer layer. The white double-headed arrows at the lower left corner of the image represent the three symmetry axes of the graphite lattice. Imaging conditions: (a) *I*_set_ = 120 pA, *V*_bias_ = −1.2 V; (b) *I*_set_ = 110 pA, *V*_bias_ = −1.45 V; (c) *I*_set_ = 140 pA, *V*_bias_ = −0.45 V; (d) *I*_set_ = 20 pA, *V*_bias_ = −0.17 V; (e) *I*_set_ = 110 pA, *V*_bias_ = −1.45 V; (f) *I*_set_ = 190 pA, *V*_bias_ = −0.4 V. For additional large-scale images, see Figure S4 in Supporting Information File 1.

Figure 4a–c shows a representative large scale STM image of the HOPG surface upon addition of a 3.8 × 10^−4^ M **BA-OC_14_** solution onto the HOPG surface covered with the ***n*-C_50_** monolayer. Surprisingly, the large-scale images reveal networks of **BA-OC_14_** adsorbed atop the ***n*-C_50_** buffer layer that are reminiscent of the ones observed on bare HOPG in the absence of the buffer layer. At this concentration of **BA-OC_14_** (≈1.9 × 10^−4^ M), we could observe all the three polymorphs atop the ***n*-C_50_** buffer layer whereas only polymorph A was formed in the absence of the buffer layer. High-resolution STM images were recorded for scrutinizing the self-assembled networks further and to determine the unit cell parameters of the three polymorphs. Figure 4d–f shows high-resolution STM images which reveal that the three structures formed atop the ***n*-C_50_** buffer layer are exactly the same as those observed when formed directly on top of the HOPG substrate. The unit cell parameters obtained for the structures formed on the buffer layer are comparable to those formed at the 1-phenyloctane–HOPG interface (Table 1).

**Figure 5:**
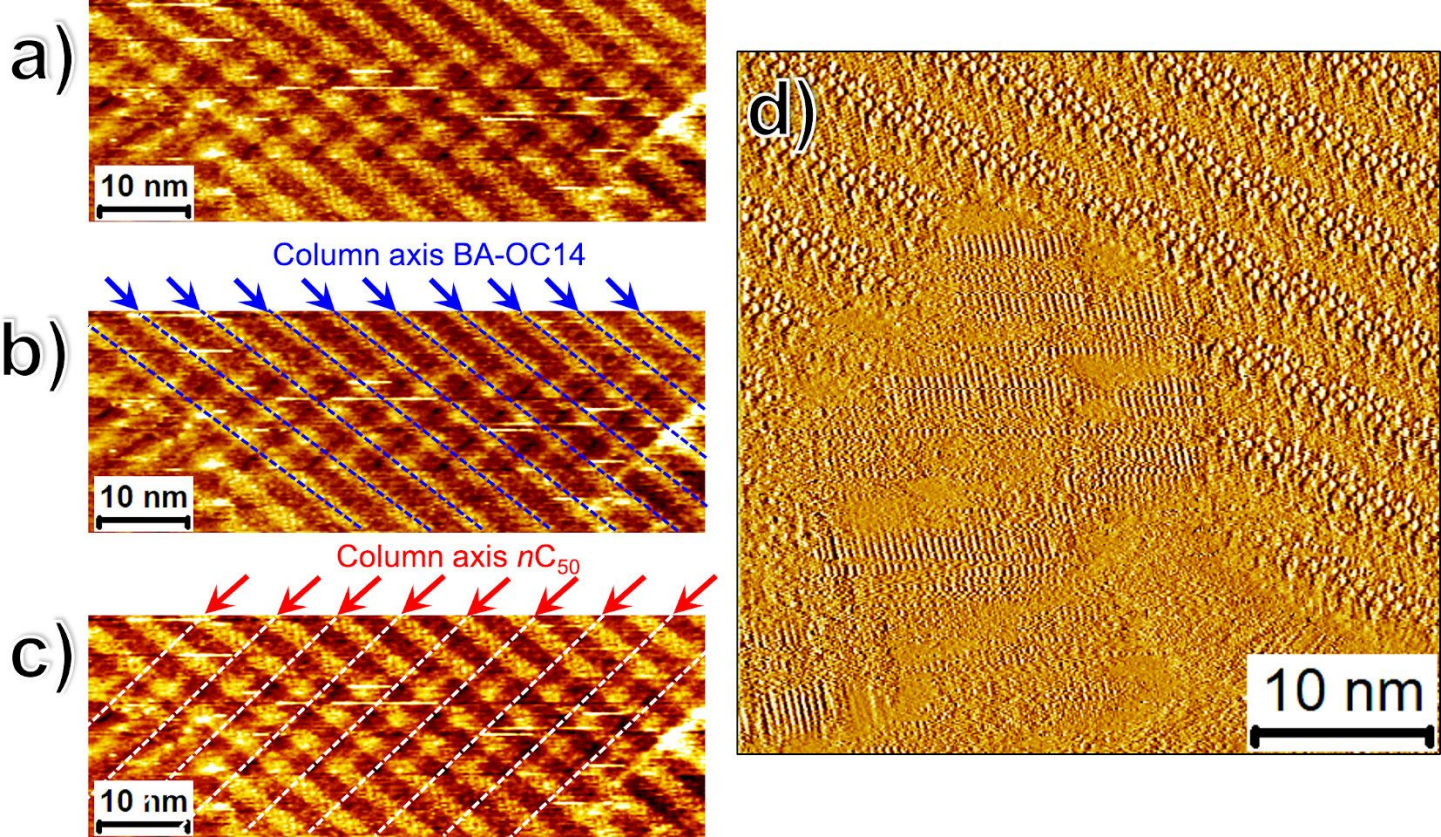
(a) STM image showing the superposition of the ***n*-C_50_** lamellae with those of **BA-OC_14_** confirming the bilayer structure of the system. Panels (b) and (c) show the same STM image with colored markers to aid the eye to identify the superimposed STM contrast arising from the columns of benzoic acid (blue dotted lines) and those of pentacontane (white dotted lines) running underneath. For the corresponding large-scale image see Figure S5 in Supporting Information File 1. Imaging conditions: *I*_set_ = 130 pA, *V*_bias_ = −0.65 V. (b) STM current image showing the alignment of the chains of the tetradecyloxy chains of **BA-OC_14_** with the long axes of ***n*-C_50_** molecules. Imaging conditions: *I*_set_ = 90 pA, *V*_bias_ = −0.17 V.

A notable feature of the high-resolution STM images presented in Figure 4d–f is that the alkyl chains of the **BA-OC_14_** molecules within the monolayers formed on top of the buffer layer still appear to be in registry with the graphite symmetry axes. This begs a question whether the **BA-OC_14_** is indeed formed on the top of the buffer layer? To confirm the bilayer formation and to rule out phase separation of the two components in 2D, we reversed the sequence of solution deposition. Thus, the solution of **BA-OC_14_** was added to the bare HOPG substrate first and the monolayer formed (polymorph A) was imaged for a few hours to ascertain full surface coverage. In the second step, ***n*-C_50_** solution was added to the surface and the STM imaging was resumed. This experiment revealed that ***n*-C_50_** completely removed the **BA-OC_14_** monolayer thus confirming that **BA-OC_14_** does not compete with ***n*-C_50_** for adsorption on the HOPG surface thereby also justifying its choice as the buffer layer. Furthermore, certain STM images clearly showed the ***n*-C_50_** lamellae running underneath the **BA-OC_14_** monolayer as displayed in Figure 5a–c. The superposition of the STM contrast of the two columns is visible in the STM image which would not be possible if the two monolayers were adsorbed side-by-side on the HOPG surface (see also Figure S5 in Supporting Information File 1). Last but not the least, the controlled nucleation experiments described below, also confirm the formation of the **BA-OC_14_** islands on top of the buffer layer. The registry of the alkyl chains of **BA-OC_14_** with the graphite lattice can be understood by considering the orientation of the **BA-OC_14_** islands with respect to the ***n*-C_50_** lamellae. As depicted in the large scale STM image provided in Figure 4c, the lamellae of polymorph A are often oriented at approximately 30° with respect to the ***n*-C_50_** lamellae (blue dashed arrows). Given that the alkyl chains of **BA-OC_14_** are oriented at an angle of 60° with respect to its lamella axis, it can be easily deduced that they are aligned along the ***n*-C_50_** molecular axis as highlighted by the white dashed arrows which indicate the long molecular axes of the ***n*-C_50_** and the **BA-OC_14_** molecules (also see Figure S6 in Supporting Information File 1). However, we also discovered that in a few cases the two molecules are not always aligned as is the case in Figure 5a–c.

For ***n*-C_50_**, the chosen optimal concentration was 1.0 × 10^−4^ M, because at this concentration we observed reasonably high average domain size and relatively low dynamics within the ***n*-C_50_** monolayer. Lowering the concentration of **BA-OC_14_** further (range ≈1.9 × 10^−4^ M to 9 × 10^−5^ M) while keeping the same concentration of ***n*-C_50_** led to exclusive formation of polymorph A. Large domains of the polymorph A were observed with very little real-time spontaneous nucleation of domains.

Considering the original goal, our pursuit for new 2D polymorphs of **BA-OC_14_** atop the ***n*-C_50_** buffer layer did not yield positive results. This also casts a doubt on the extent of the influence of the substrate lattice on the assembly of alkoxybenzoic acids as originally proposed by us [45]. While the observation of similar polymorphs on top of the buffer layer does not necessarily rule out the role of substrate lattice on the assembly, it is certainly not the sole explanation for the peculiar network structure of polymorph A. At this juncture, we believe that the formation of the characteristic kinked lamellae in the case of 4-alkoxybenzoic acids is a result of a tendency to achieve close-packed assemblies together with the contribution of various other factors, the influence of the substrate lattice being possibly one of them. We have recently reported on similar behavior in the case of brominated alkoxybenzenes where a brick-wall type structure was ascribed to a tendency to form close packed networks since the formation of kinks/fractures within the monolayer allowed higher packing densities in the monolayers [47].

While studying the self-assembly of **BA-OC_14_** on the buffer layers, we discovered a number of interesting aspects of this peculiar interface. As anticipated, the mobility of **BA-OC_14_** on the buffer layers was significantly higher than that at the 1-phenyloctane–HOPG interface. It was possible to observe the nucleation, the growth as well as the desorption of molecular domains (see Figure S7 in the Supporting Information File 1) which was otherwise not possible for monolayers adsorbed directly on the graphite surface. In the following section we describe our attempts to observe such dynamic phenomena and to induce nucleation of **BA-OC_14_** domains using the STM tip.

### STM tip-induced nucleation on-command atop ***n*-C_50_** buffer layer

**Figure 6:**
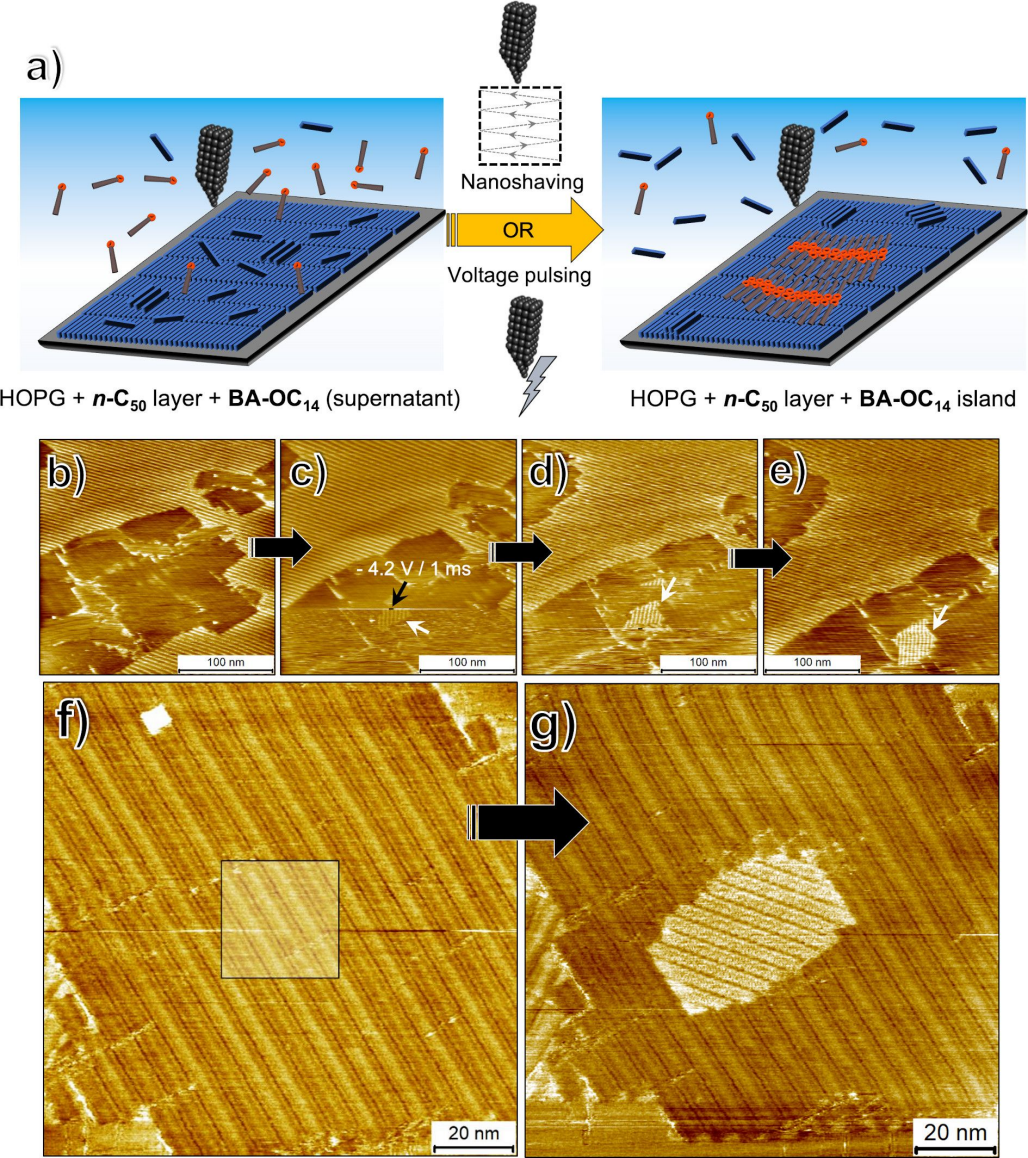
On-command nucleation of **BA-OC_14_** islands on top of the ***n*-C_50_** buffer layer. (a) Schematic for the STM-induced nucleation process. (b–e) Sequential STM images where the nucleation of **BA-OC_14_** islands was achieved using application of voltage pulses to the STM tip. The black arrow in (c) shows the point at which a −4.2 V (1 ms) pulse was applied to the STM tip. The white arrow shows the island of **BA-OC_14_** that nucleated immediately after the pulse. As evident in panels (d,e) this island grew in subsequent scans. (f,g) Sequential STM images showing nucleation on-command by bringing the STM tip closer to the surface and scanning at high tunneling current. The square highlighted in (f) was scanned at −0.05 V and 0.5 nA. After this, the original image size was restored by zooming out. As evident from (g) a new island of **BA-OC_14_** is nucleated approximately in the area which was manipulated in the previous scan. Imaging conditions: (b–e) *I*_set_ = 130 pA, *V*_bias_ = −1.3 V; (f,g) *I*_set_ = 140 pA, *V*_bias_ = −0.7 V.

During the course of this investigation, we uncovered that it is possible to enforce the nucleation of small **BA-OC_14_** islands on the buffer layers using the STM tip. Such “on-command” nucleation was achieved using two different stimuli, namely by applying short voltage pulses to the STM tip and by scanning the surface of the buffer layer at higher than normal tunneling currents (Figure 6a). Application of voltage pulses to the STM tip while imaging the ***n*-C_50_** buffer layer (containing **BA-OC_14_** in the supernatant) lead to nucleation of small islands of polymorph A of **BA-OC_14_**. The domain size was typically around 20 × 20 nm. These domains were found to either grow or shrink upon subsequent STM imaging of the same area. Figure 6b–e shows one such pulse-induced on-command nucleation event. A voltage pulse with a magnitude of −4.2 V lasting 1 ms was applied onto an ‘empty’ region of the ***n*-C_50_** buffer layer (marked by an arrow in Figure 6c) which did not have any adsorbed domain of **BA-OC_14_**. A **BA-OC_14_** domain can be seen to appear right after the voltage pulse was applied (white arrow, Figure 6c) and was found to grow in the subsequent scans (white arrows, Figure 6d,e). Such pulse-induced nucleation was possible to carry out by selecting any area of the buffer layer which did not have a **BA-OC_14_** domain. We also systematically examined the influence of the magnitude and the duration of the voltage pulse on the nucleation events. This exercise revealed that a pulse width of 1 ms and a minimum required pulse height of −3.3 V was necessary for inducing the nucleation of **BA-OC_14_** domains. The outcome of the pulse-induced nucleation experiment remained the same irrespective of the sign of the pulse.

Scanning small areas of the ***n*-C_50_** buffer layer (with **BA-OC_14_** supernatant) at higher tunneling currents and lower bias (typically −0.05 V and 0.5 nA) provided better control over the on-command nucleation process. These parameters are used for obtaining the resolution at which the graphite lattice is visible. Figure 6f shows an STM image of the buffer layer. The area highlighted by the square (30 nm × 30 nm) was scanned using the aforementioned parameters. In the subsequent scan, the parameters were changed to normal imaging parameters and the size of the scan was restored to the original (zoomed out). The subsequent scan presented in Figure 6g reveals the presence of a small domain of **BA-OC_14_** in the area scanned at the different tunneling parameters. It can also be noticed that the lamellae of the new domain follow the same registry conditions (≈30° with respect to the column axis of ***n*-C_50_**) as described earlier. This means that the tetradecyloxy chains of **BA-OC_14_** are aligned with the alkane molecules underneath. We note that the ‘nucleation on command’ experiments always furnished polymorph A.

Quite naturally, one could suspect that the observed nucleation occurs on top of the graphite surface, because under the scanning parameters used, the STM tip is pushed very close to the substrate allowing possible removal of the buffer layer. If this was the case, one would observe gradual removal of the nucleated domain of **BA-OC_14_** by ***n*-C_50_** molecules. This was not observed. In fact, in some cases, after inducing the nucleation in this fashion, the newly formed **BA-OC_14_** domain was found to grow rapidly (Figure S8 in Supporting Information File 1). If this would happen in a hypothetical scenario where the **BA-OC_14_** domain nucleated directly on HOPG, it would require the displacement of ***n*-C_50_** molecules by **BA-OC_14_** molecules which was never observed during the independent control experiments. Similar to the pulse-induced process, the scanning-induced nucleation could also be stimulated a number of times although in some instances (minority cases) no nucleation of **BA-OC_14_** domain was observed.

So, what is the mechanism behind the on-command nucleation of **BA-OC_14_** on the buffer layers? We propose that the stimuli provided, namely scanning at higher currents and the voltage pulsing, removes the residual layer of ***n*-C_50_** molecules interacting with the monolayer of ***n*-C_50_** adsorbed on the surface. Given that the concentration of ***n*-C_50_** in solution is well above than that required to form a monolayer, it is not unreasonable to consider that there exist excess molecules of ***n*-C_50_** that interact with the preformed buffer layer. ‘Blasting’ the surface of the buffer layer with a voltage pulse or ‘sweeping’ it by bringing the tip closer to the surface removes these excess ***n*-C_50_** molecules thereby allowing the nucleation of **BA-OC_14_** islands which then subsequently grow. The observed relative concentration dependency of nucleation and growth also suggests that a higher ***n*-C_50_** concentration ‘blocks’ the nucleation of **BA-OC_14_** on top of the buffer layer.

## Conclusion

New structures, whether in bulk or on surfaces, are found as long as one keeps looking for them by continuously adjusting experimental conditions that affect (2D) crystallization. Keeping this generally accepted perception in mind, we had set out to alter an important factor that is known to influence self-assembly on solid surfaces, namely the influence of the substrate lattice. We used a monolayer of ***n*-C_50_** as a ‘new’ substrate for the self-assembly of **BA-OC_14_** at the solution–solid interface and anticipated that, given the different lattice constant of the ***n*-C_50_** layer compared to graphite, we would create possibilities for the formation of new polymorph(s). The self-assembly experiments using the buffer layer however revealed that **BA-OC_14_** forms the same structural polymorphs in the presence and in the absence of the buffer layer. This indicates that the peculiar network formation, at least in the case of polymorph A, cannot be solely explained by the influence of the substrate lattice. We hypothesize that in the case of alkoxybenzoic acids, a tendency to form close-packed networks supersedes other factors.

The buffer layer however provided a unique platform for self-assembly experiments where the nucleation, growth and ripening of the self-assembled monolayer could be monitored in a more controlled fashion compared to that on the surface of HOPG. The on-command nucleation demonstrated here is an exciting approach to study nucleation of typical systems with precise control on the size and shape of the domains that can be formed using the STM tip. This approach holds a promise as a new platform for investigating elementary stages of self-assembly processes. Despite the lack of discovery of new polymorphs in the present case, the buffer layer strategy could prove fruitful towards identification of new structures in future.

## Experimental

***n*-C_50_** was obtained from Sigma-Aldrich (purity ≥97%) and used without further purification. **BA-OC_14_** was synthesized using a protocol previously reported by us [45]. Stock solutions of ***n*-C_50_** (1.0 × 10^−3^ M) and **BA-OC_14_** (7.7 × 10^−4^ M) were prepared by dissolving appropriate amounts of solid materials in 1-phenyloctane (Sigma-Aldrich, >99%). The stock solutions were diluted further with 1-phenyoctane to make concentration series. All STM experiments were performed at room temperature (21–23 °C) using a PicoLE or a PicoSPM (Agilent) machine operating in constant-current mode with the tip immersed in the supernatant liquid. STM tips were prepared by mechanically cutting a Pt/Ir wire (80%/20%, diameter 0.2 mm). Prior to imaging, a drop of solution was placed onto a freshly cleaved surface of highly oriented pyrolytic graphite (HOPG, grade ZYB, Advanced Ceramics Inc., Cleveland, USA). For experiments involving the ***n*-C_50_** buffer layer, both sequential deposition and premixing protocols were used. For sequential deposition, a solution of ***n*-C_50_** was first applied to a freshly cleaved graphite surface. The STM imaging was carried out to ensure full surface coverage of the buffer layer. After this, a drop of **BA-OC_14_** solution was applied and the imaging was resumed. The STM experiments were repeated in 2–3 sessions using different tips to check for reproducibility and to avoid experimental artefacts, if any. For voltage pulsing experiments described here, the pulses were applied during the line scan. Each voltage pulse was applied to the substrate after retracting the STM tip at a certain distance (around 1 nm) from the surface. The feedback loop was turned off to maintain the separation between the tip and the sample during the period of voltage pulse in order to avoid the tip crash onto the surface. The software used for STM imaging does not log the tunneling current reached during each pulse. For analysis purposes, recording of a monolayer image was followed by imaging the graphite substrate underneath it under the same experimental conditions, except for increasing the current and lowering the bias. The images were corrected for drift via Scanning Probe Image Processor (SPIP) software (Image Metrology ApS), using the recorded graphite images for calibration purposes, allowing a more accurate unit cell determination. The unit cell parameters were determined by examining at least 4 images and only the average values are reported. The images are Gaussian filtered and/or correlation averaged. The imaging parameters are indicated in the figure caption: tunneling current (*I*_set_), and sample bias (*V*_bias_). The molecular models were built using HyperchemTM 8.0.1 program.

## Supporting Information

File 1 Additional STM data.
