# Supplementary material for: Growth of a self-assembled monolayer decoupled from the substrate: nucleation on-command using buffer layers
Source: Beilstein J Nanotechnol. 2020 Sep 1;11:1291–302. doi: 10.3762/bjnano.11.113 (PMC7476593; doi:10.3762/bjnano.11.113)
Supplement: File 1 — Additional STM data. [file Beilstein_J_Nanotechnol-11-1291-s001.pdf]

BEILSTEIN JOURNAL OF NANOTECHNOLOGY

Supporting Information

for

Growth of a self-assembled monolayer decoupled from the substrate: nucleation on-command using buffer layers

Robby Reynaerts, Kunal S. Mali and Steven De Feyter

Beilstein J. Nanotechnol. **2020,** *11,* 1291–1302. doi:10.3762/bjnano.11.113

Additional STM data

License and Terms: This is a supporting information file under the terms of the Creative Commons Attribution License (https://creativecommons.org/licenses/by/4.0). Please note that the reuse, redistribution and reproduction in particular requires that the authors and source are credited.
The license is subject to the *Beilstein Journal of Nanotechnology* terms and conditions: (https://www.beilstein-journals.org/bjnano)

Contents:

1. Additional large scale STM images of the **BA-OC$_{14}$** polymorphs observed at the 1-phenyloctane–HOPG interface (Figure S1).
2. Magnified version of high-resolution STM images and molecular models presented in Figure 2 in the main text (Figure S2).
3. Sequential STM images showing the dynamic rearrangement in the monolayer of ***n*-C$_{50}$** at lower concentrations (Figure S3).
4. Additional STM images for the domains of polymorph A, B and C nucleated on top of the ***n*-C$_{50}$** buffer layer (Figure S4).
5. STM images showing the superposition of the STM contrast of the bilayer showing overlapping columns (Figure S5)
6. STM images showing the alignment of the benzoic acid polymorphs with the ***n*-C$_{50}$** buffer layer (Figure S6).
7. STM images showing the nucleation, growth and desorption of **BA-OC$_{14}$** polymorphs on top of the ***n*-C$_{50}$** buffer layer (Figure S7).
8. Sequential STM images showing the rapid growth of the **BA-OC$_{14}$** domain obtained after nanoshaving (Figure S8).
9. STM image showing a benzoic acid island formed on top of the buffer layer with a line profile (Figure S9).

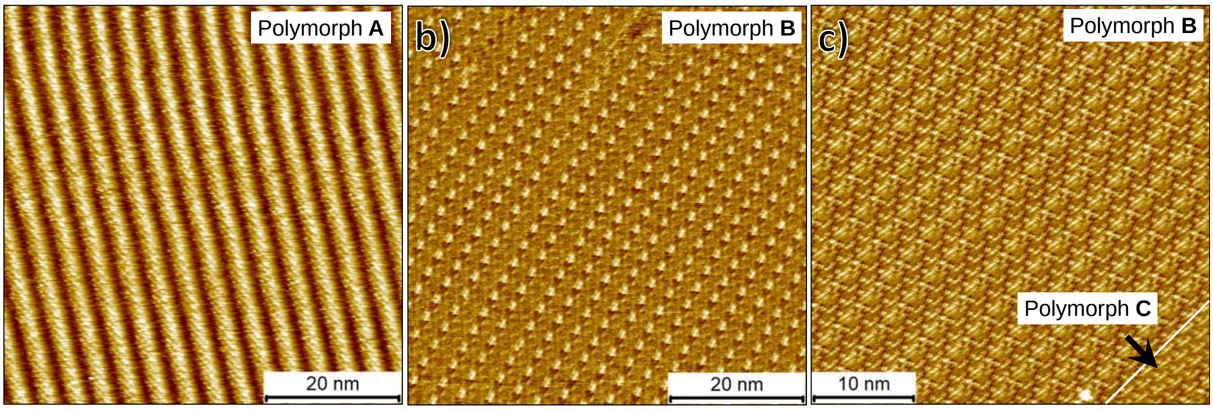

Figure S1: Additional large scale STM images of the three polymorphs of **BA-OC$_{14}$** observed at the 1-phenyloctane/HOPG interface. [**BA-OC$_{14}$**] = 9×10^{-5} M. Imaging conditions: (a) I_{set} = 140 pA, V_{bias} = -0.7 V. (b) I_{set} = 160 pA, V_{bias} = −1.45 V. (c) I_{set} = 150 pA, V_{bias} = 0.55 V.

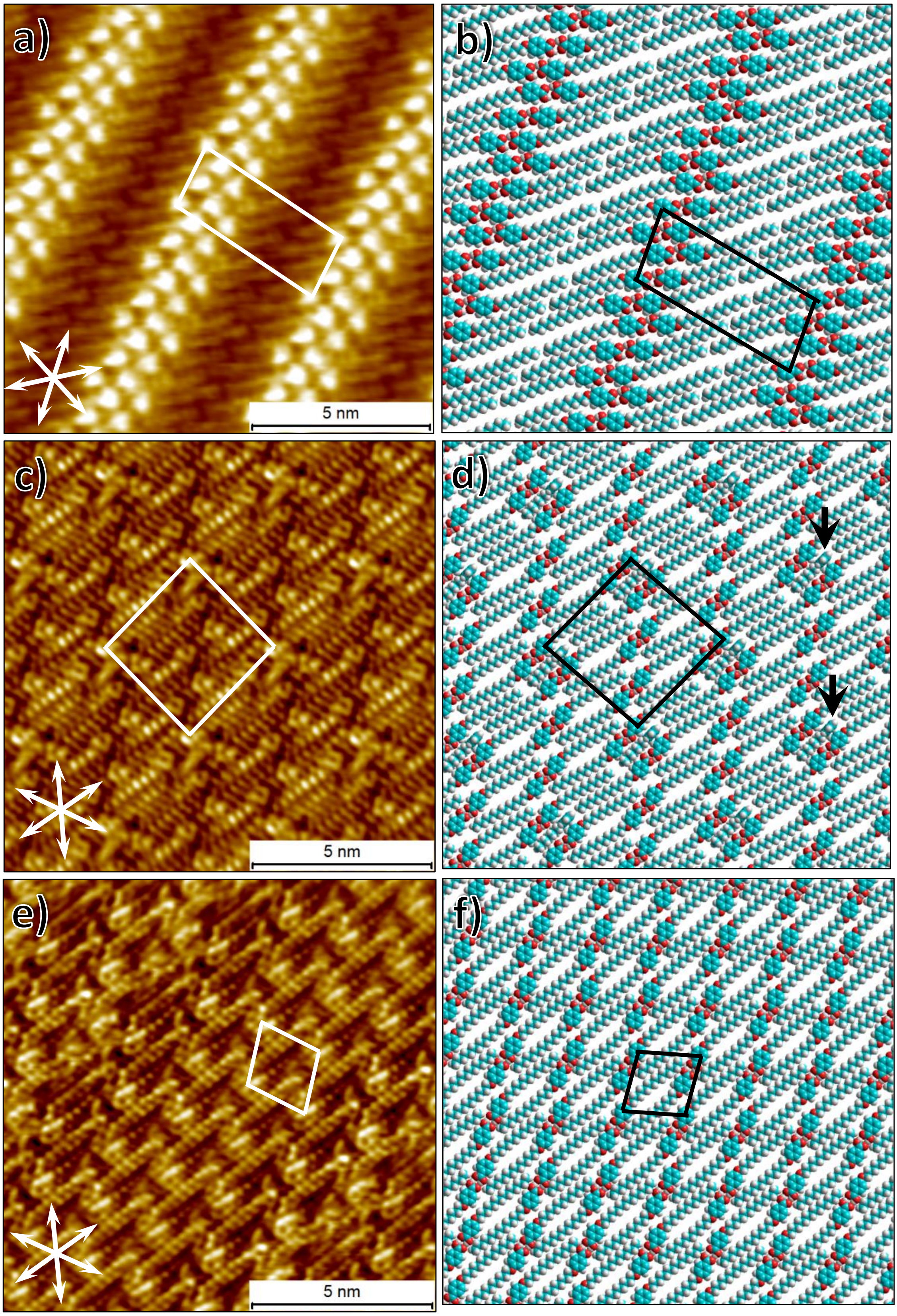

Figure S2: Magnified version of Figure 2 provided in the main text highlighting the high-resolution STM images and the corresponding molecular models.

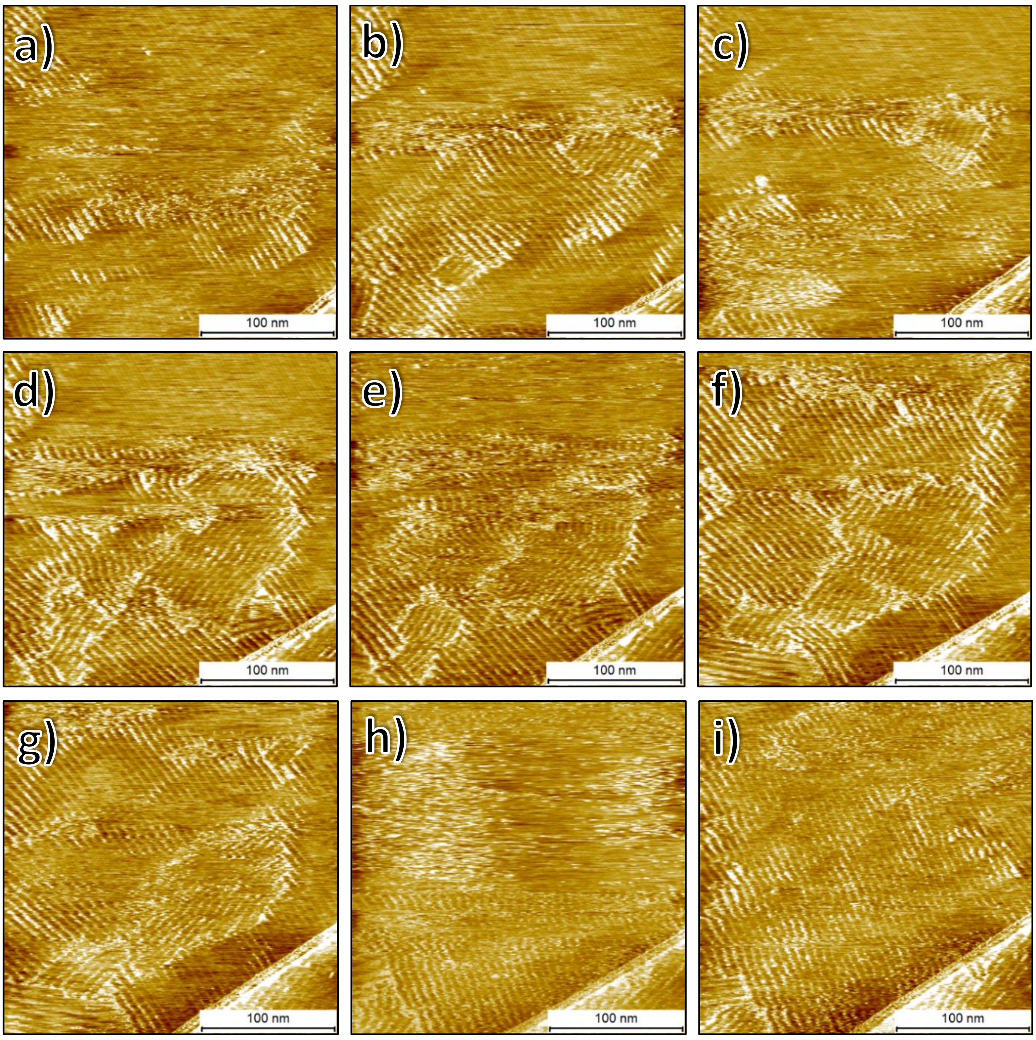

Figure S3: Sequential STM images showing the highly dynamic behavior of ***n*-C$_{50}$** at 7.0 × 10^{-6} M at the 1-phenyloctane–HOPG interface. With each scan significant rearrangement in the domain structure is visible. The sample was annealed at 50 °C for 15 min and afterwards rinsed with 1-phenyloctance. Imaging conditions: I_{set} = 160 pA, V_{bias} = −0.7 V.

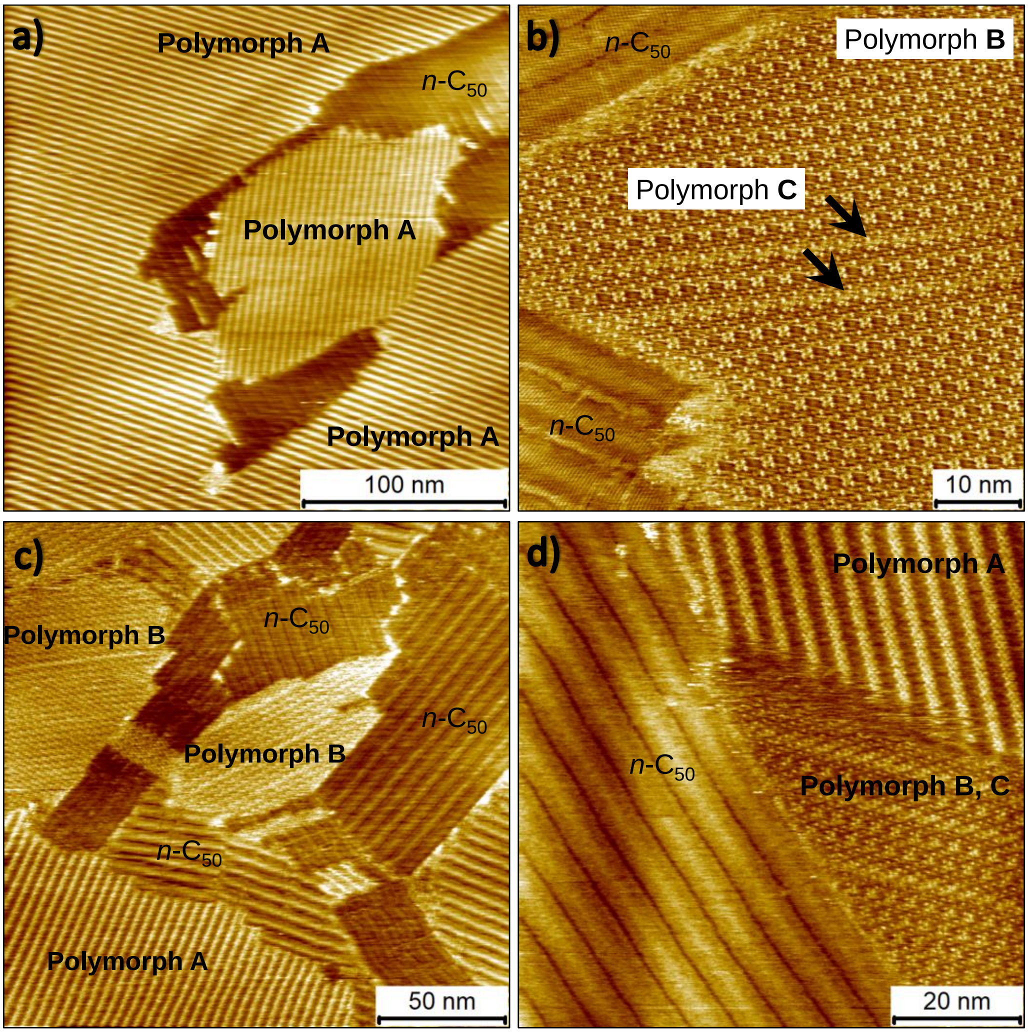

Figure S4: Additional STM images for the domains of polymorph **A, B** and **C** nucleated on top of the ***n*-C$_{50}$** buffer layer. Imaging conditions: (a) I_{set} = 130 pA, V_{bias} = −1.3 V. (b) I_{set} = 190 pA, V_{bias} = −0.4 V. (c) I_{set} = 140 pA, V_{bias} = −0.65 V. (d) I_{set} = 140 pA, V_{bias} = −0.45 V.

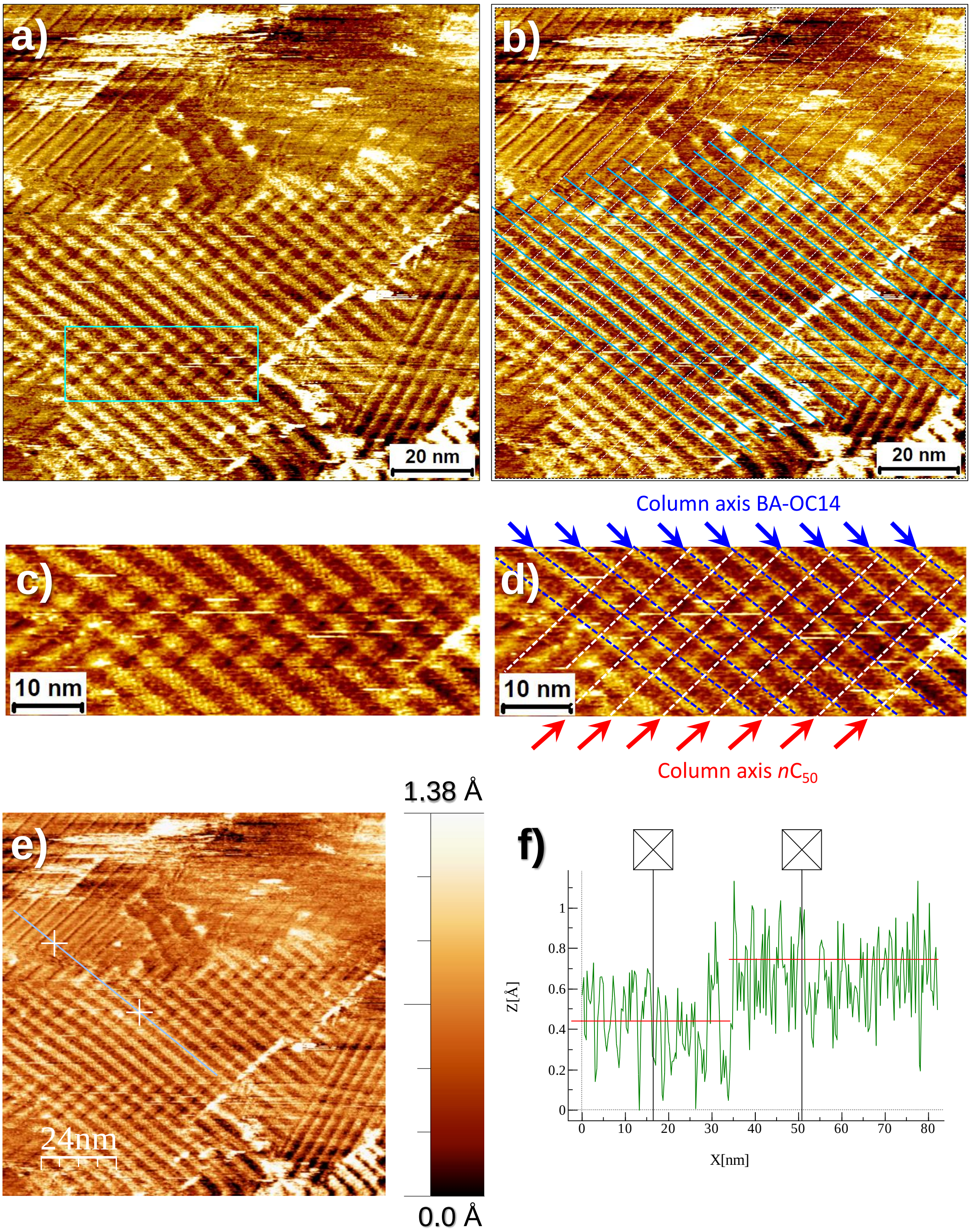

Figure S5: (a) Large scale STM image showing peculiar STM contrast that shows overlap of features of the columns of **BA-OC$_{14}$** and ***n*-C$_{50}$**. (b) Same STM image as in (a) but with overlaid colored markers for identifying the columns of **BA-OC$_{14}$** (light blue) and ***n*-C$_{50}$** (white). (c) Digital zoom of the area marked by cyan rectangle in (a). (d) The same zoom with overlaid markers. (e, f) Line profile across the molecular columns of **BA-OC$_{14}$** and ***n*-C$_{50}$**. Imaging conditions: I_{set} = 130 pA, V_{bias} = −0.65 V.

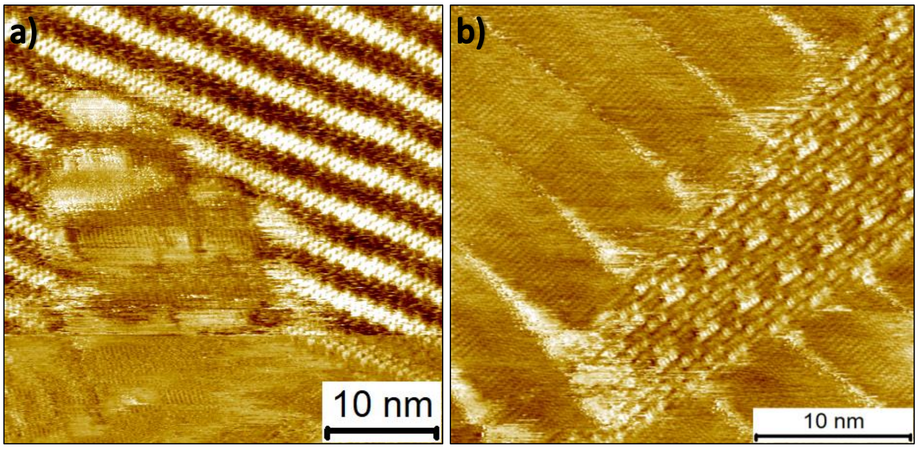

Figure S6: STM images showing the approximate alignment of the alkoxy chains of polymorphs A and B of **BA-OC$_{14}$** with the alkyl chains of ***n*-C$_{50}$** at the 1-phenyloctane–HOPG interface. Imaging conditions: (a) I_{set} = 90 pA, V_{bias} = –0.17 V. (b) I_{set} = 80 pA, V_{bias} = –0.95 V.

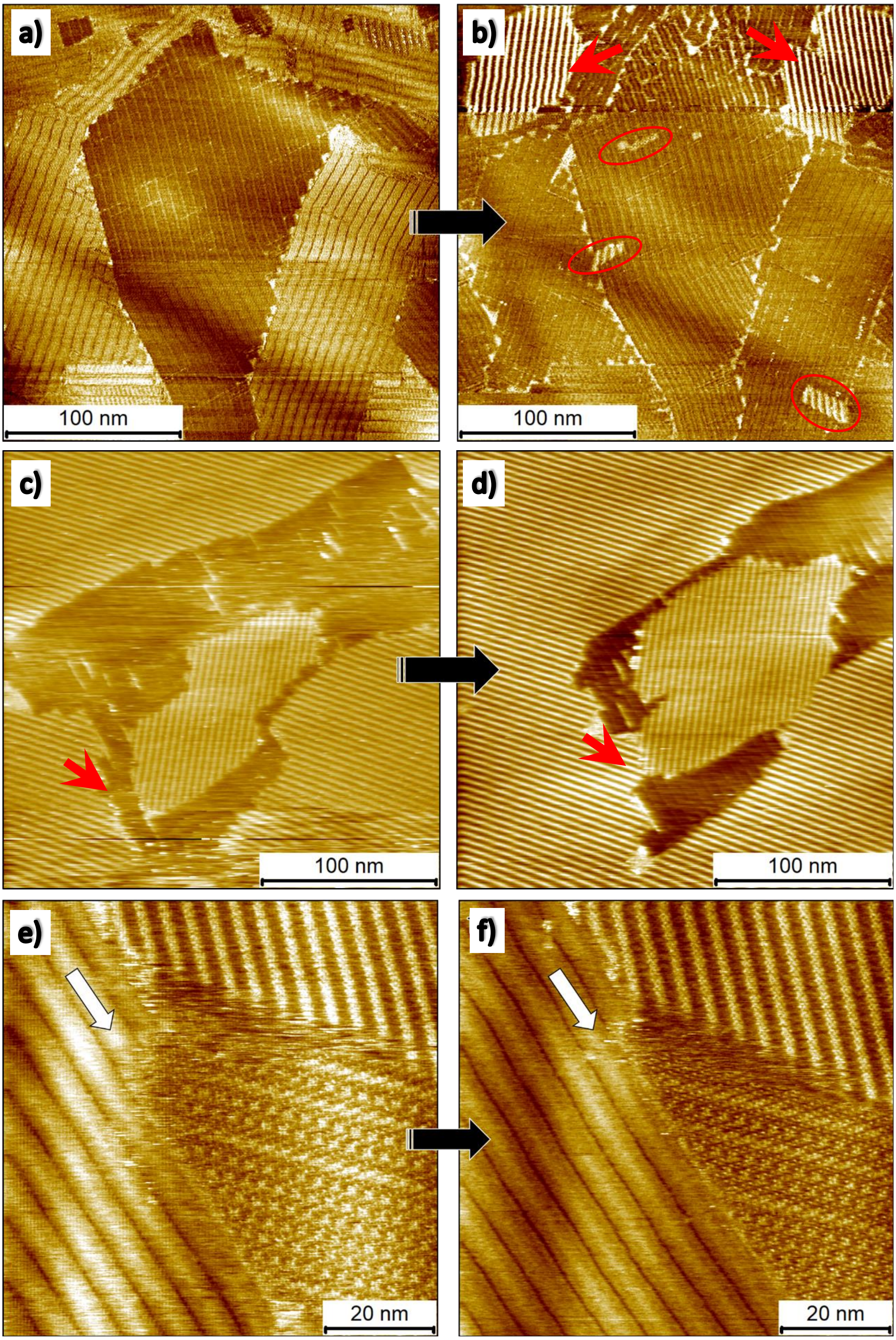

Figure S7: STM images showing the nucleation (a,b), growth (c,d) and desorption (e,f) of **BA-OC$_{14}$** on top of the ***n*-C$_{50}$** buffer layer at the 1-phenyloctane–HOPG interface. (a,b) I_{set} = 130 pA, V_{bias} = −0.85 V (c,d) I_{set} = 130 pA, V_{bias} = −1.3 V. (e,f) I_{set} = 140 pA, V_{bias} = −0.45 V.

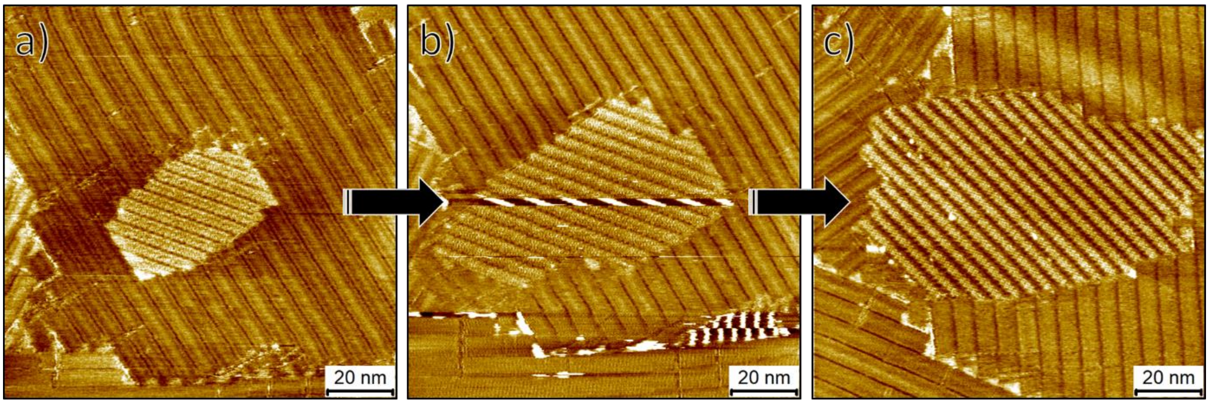

Figure S8: Rapid growth of **BA-OC$_{14}$** domain on top of the ***n*-C$_{50}$** buffer layer at the 1-phenyloctance–HOPG interface after nanoshaving-induced nucleation.(a) I_{set} = 140 pA, V_{bias} = -0.7 V (b) I_{set} = 170 pA, V_{bias} = -0.55 V (c) I_{set} = 90 pA, V_{bias} = -0.55 V.

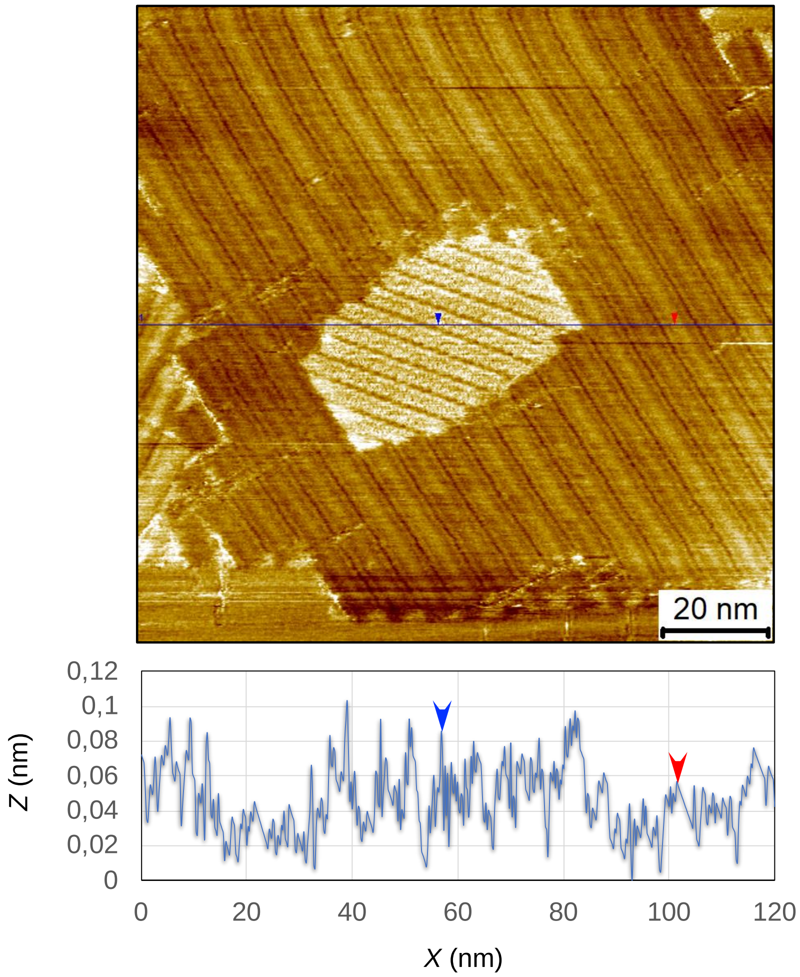

Figure S9: STM image showing an island of **BA-OC$_{14}$** monolayer formed on top of the buffer layer with a line profile across the island. Note that height in STM images is relative (thus the *Y* axis does not represent absolute numbers) and is a convolution of electronic and topographic effects. This image is the same as the one presented in Figure 6g in the main text.
